# Genetically encoded tools for in vivo G‐protein‐coupled receptor agonist detection at cellular resolution

**DOI:** 10.1002/ctm2.1124

**Published:** 2022-11-29

**Authors:** Kayla E. Kroning, Wenjing Wang

**Affiliations:** ^1^ Life Sciences Institute, University of Michigan Ann Arbor Michigan USA; ^2^ Department of Chemistry University of Michigan Ann Arbor Michigan USA

**Keywords:** genetically encoded sensor, GPCR, neuromodulation, neurotransmission

## Abstract

G‐protein‐coupled receptors (GPCRs) are the most abundant receptor type in the human body and are responsible for regulating many physiological processes, such as sensation, cognition, muscle contraction and metabolism. Further, GPCRs are widely expressed in the brain where their agonists make up a large number of neurotransmitters and neuromodulators. Due to the importance of GPCRs in human physiology, genetically encoded sensors have been engineered to detect GPCR agonists at cellular resolution in vivo. These sensors can be placed into two main categories: those that offer real‐time information on the signalling dynamics of GPCR agonists and those that integrate the GPCR agonist signal into a permanent, quantifiable mark that can be used to detect GPCR agonist localisation in a large brain area. In this review, we discuss the various designs of real‐time and integration sensors, their advantages and limitations, and some in vivo applications. We also discuss the potential of using real‐time and integrator sensors together to identify neuronal circuits affected by endogenous GPCR agonists and perform detailed characterisations of the spatiotemporal dynamics of GPCR agonist release in those circuits. By using these sensors together, the overall knowledge of GPCR‐mediated signalling can be expanded.

## Graphical abstract

G‐protein‐coupled receptor (GPCR) agonist sensors utilise downstream GPCR signalling events, such as GPCR conformational changes and protein binding, to give a measurable sensor readout.

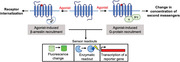

## 1 INTRODUCTION

### 1.1 Importance of GPCR signalling

**FIGURE 1:**
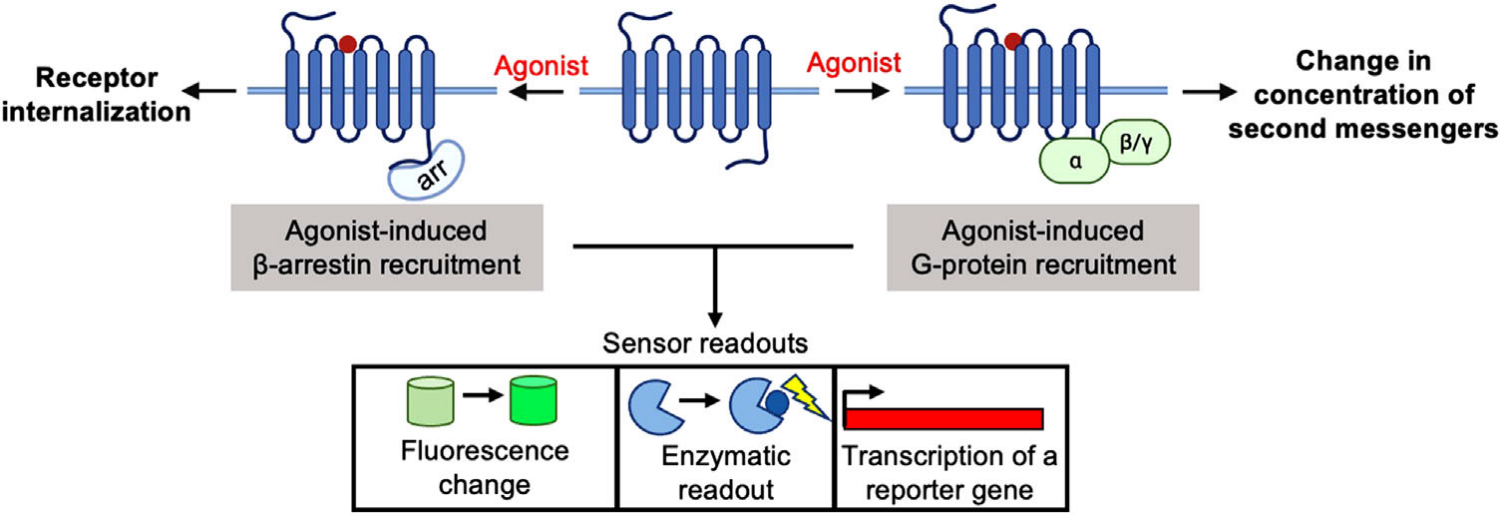
Schematic of G‐protein‐coupled receptor (GPCR) signalling cascade. Agonist binding causes a conformational change in the GPCR, which recruits G‐proteins and/or β‐arrestin to the GPCR. G‐protein binding leads to a change in concentration of second messengers and β‐arrestin binding causes internalisation of the GPCR. These agonist‐induced changes can be utilised in sensors, where they can cause the change of fluorescence, an enzymatic readout, or the transcription of a reporter gene.

G‐protein‐coupled receptors (GPCRs) are seven‐transmembrane domain receptors responsible for modulating an abundance of physiological processes, including sensation,^1^ cognition,^2^ muscle contraction^3^ and metabolism.^4^ Consisting of more than 800 receptor types, GPCRs are the largest family of membrane receptors in the human body.^5^ Once activated by external stimuli including small molecule neurotransmitters, peptides, lipids, ions and light, the GPCR undergoes a conformational change, which allows G‐protein and/or β‐arrestin binding.^6^, ^7^, ^8^ G‐protein binding catalyses the activation of a downstream signalling cascade, where, depending on the G‐protein subtype, can cause a change in the intracellular concentration of second messengers, such as cAMP, IP_3_, Ca^2+^ and diacylglycerol, while β‐arrestin binding can cause internalisation of the GPCR^9^, ^10^ (Figure 1).

GPCR signalling malfunction is involved in a large number of diseases, such as heart disease,^11^ depression,^12^ dementia,^13^ Alzheimer's disease,^13^ Parkinson's disease^13^ and Huntington's disease.^13^ Approximately 35% of drugs approved by the U.S. Food and Drug Administration (FDA) target GPCRs.^14^ Additionally, many neuromodulators and neurotransmitters are GPCR agonists, such as glutamate, γ‐aminobutyric acid (GABA), acetylcholine, serotonin, dopamine and various neuromodulating peptides. GPCRs, therefore, play critical roles in modulating the activity of the nervous system. GPCR activity is mainly regulated by the spatiotemporally regulated release of endogenous agonists. Therefore, there is particular interest in studying the spatiotemporal dynamics of endogenous GPCR agonists to understand how the precise timing and spatial localisation of GPCR activation affects the downstream signalling cascade and overall biological response.

### 1.2 Overview of genetically encoded sensors for detecting endogenous GPCR agonists

To gain information about the spatiotemporal dynamics of GPCR activation in biological models, a variety of genetically encoded sensors have been engineered to detect GPCR agonists. Bioanalytical methods, such as microdialysis^15^ and fast scanning cyclic voltammetry,^16^, ^17^, ^18^, ^19^ have been useful for GPCR agonist detection; however, genetically encoded GPCR sensors provide unique advantages. Genetic encoding offers cell‐type specificity when imaging in the animal brain and can image at cellular or even subcellular resolution. Genetically encoded GPCR sensors, therefore, can be used to determine the precise localisation and release dynamics of GPCR agonist release.

Genetically encoded sensor designs are mainly based on the agonist‐induced conformational change of the third intracellular loop, G‐protein or β‐arrestin binding, or the change of concentration of second messengers (Figure 1). The readout of these sensors can be the activation of a fluorescent protein, an enzymatic reaction, or the transcription of a reporter gene.

**FIGURE 2:**
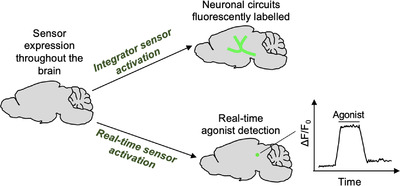
Integrators versus real‐time sensors. Integrators leave a permeant mark in the cells exposed to G‐protein‐coupled receptor (GPCR) agonists, so large brain regions can be analysed at cellular resolution. Real‐time sensors can be used to observe the real‐time agonist‐induced neuronal activity.

Genetically encoded GPCR sensors can be divided into two main categories: those that offer real‐time information about GPCR agonist‐induced activation and those that integrate the GPCR agonist signal, leaving a permanent quantifiable signal over a large region of the brain (Figure 2). Real‐time sensors offer important information about the signalling dynamics of GPCRs and integrators can permanetely mark neurons exposed to GPCR agonists for further analysis. In this review, we will discuss the recent advancement of genetically encoded real‐time sensors and integrators for detecting GPCR agonists. We will also discuss how these two categories of sensors can be used together to provide detailed information about the spatiotemporal release of GPCR agonists in a whole brain at cellular resolution.

## 2 Genetically encoded real‐time sensors

A variety of genetically encoded real‐time sensors for detecting GPCR agonists have been recently developed using five major strategies: (1) intra‐ or intermolecular resonance energy transfers; (2) circularly permuted green fluorescent protein (cpGFP) insertion into periplasmic binding proteins; (3) cpGFP inserted into the third intracellular loop of a GPCR; (4) translocation assays; and (5) detection of second messengers following GPCR activation. Real‐time sensors are beneficial for studying the signalling kinetics of neurotransmitters and neuromodulators; therefore, these sensors benefit from having fast on and off rates. In this review, we will focus on the various designs of real‐time sensors, but we will not discuss in detail the kinetics of real‐time sensors. Readers can see reference^20^ for time constant values of many of the sensors discussed here.

### 2.1 FRET‐ and BRET‐based sensors

Fluorescence resonance energy transfer (FRET)‐based GPCR agonist sensors use a donor and acceptor fluorescent protein, where the donor's emission spectrum overlaps with the acceptor's excitation spectrum. FRET efficiency is inversely proportional to *r* raised to the sixth power, where *r* is the distance between the donor and acceptor fluorophore. Therefore, FRET efficiency can be highly sensitive to GPCR activation‐dependent conformational change. The FRET fluorescent protein pairs can either be placed on different locations on the same GPCR (intramolecular FRET) or one protein can be fused to the GPCR and the other can be fused to a protein interactor, such as a G‐protein mimic or β‐arrestin (intermolecular FRET). Bioluminescence resonance energy transfer (BRET) is similar to FRET, except that a bioluminescent protein acts as the energy donor to activate the fluorescent protein acceptor.^21^

Here, we will mainly discuss the FRET‐ and BRET‐based sensors that can potentially be used for detection of GPCR agonists in vivo at cellular resolution. For a more comprehensive review of FRET‐ and BRET‐based GPCR sensors, see reference.^21^ We will divide FRET and BRET sensors into four main categories: (1) those that detect G‐protein activation; (2) those that detect β‐arrestin recruitment; (3) those that detect intramolecular GPCR conformational changes; and (4) those that detect the conformational change of binding proteins.

#### 2.1.1 BRET and FRET sensors based on G‐protein binding and activation

**FIGURE 3:**
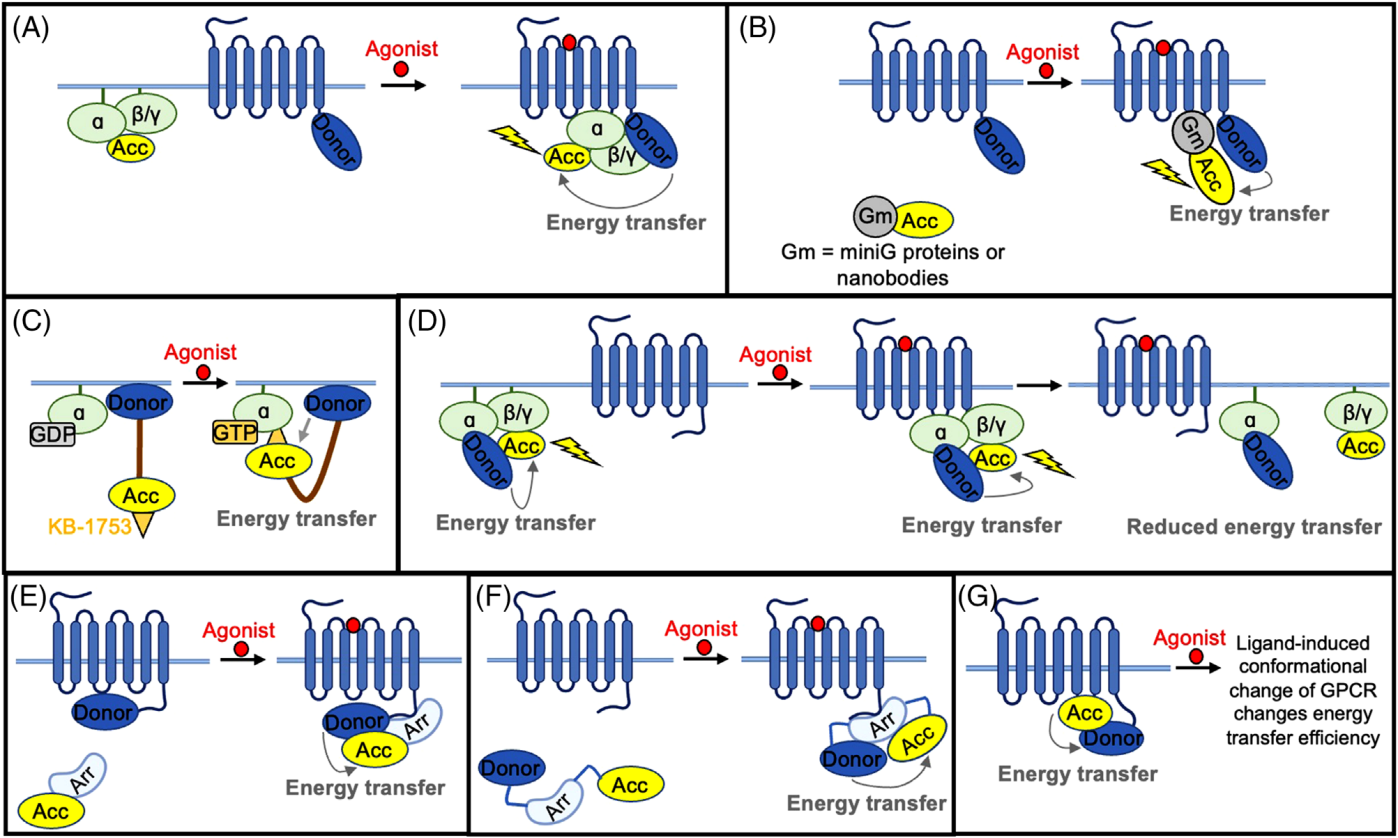
Fluorescence resonance energy transfer (FRET) and bioluminescence resonance energy transfer (BRET)‐based sensors. (A) Resonance energy transfer (RET)‐based sensor, where one RET pair is attached to the Gα‐protein and the other is attached to the G‐protein‐coupled receptor (GPCR). GPCR activationrecruits the G‐proteins, increasing the energy transfer efficiency. Acc, RET acceptor. (B) RET‐based sensor, where one RET pair is attached to the Gα‐protein mimic, such as a miniG protein or a nanobody, and the other is attached to the GPCR. GPCR activation recruits the G‐protein mimic, increasing the energy transfer efficiency. (C) Schematic of BERKY. BRET pairs are separated by an ER/K α‐helix linker, where the acceptor is at the end of the linker and the donor is at the start of the linker, fused to the membrane. KB‐1753, a peptide that binds to the active Gα‐protein, is attached to the acceptor pair. Agonist results in G‐protein activation which results in KB‐1753 binding and an increase in energy transfer efficiency. (D) RET‐based sensor, where one RET pair is attached to the Gα‐protein and the other is attached to the Gβ‐ and γ‐proteins. GPCR activation causes the distance to increase between the Gα and Gβγ proteins, decreasing the energy transfer efficiency. (E) RET‐based sensor, where one RET pair is attached to β‐arrestin and the other is attached to the GPCR. GPCR activation recruits β‐arrestin, increasing the energy transfer efficiency. (F) RET‐based sensor, where RET pairs are attached to either terminus of β‐arrestin. Agonist activation of the GPCR causes a conformational change in β‐arrestin, where there is an increase in energy transfer efficiency. (G) RET‐based sensor, where one RET pair is attached to the third intracellular loop of the GPCR and the other is attached to the GPCR's C‐terminal tail. Agonist‐induced activation causes a conformational change in the GPCR, thereby changing the energy transfer efficiency.

To detect G‐protein activation, a bioluminescent protein, such as *Renilla* luciferase (Rluc) or the smaller luciferase enzyme, Nano Luciferase (NLuc),^22^ fused to a GPCR can act as the BRET donor and a fluorescent protein, such as Venus or yellow fluorescent protein (YFP), fused to a Gα‐protein or a Gα‐mimic can act as the BRET acceptor. When the GPCR is activated by its agonist, the Gα‐protein/mimic binds to the intracellular portion of the GPCR, thereby bringing the two BRET pairs into proximity where energy transfer can take place (Figure 3A,B). BRET‐based GPCR sensors have been used to detect GPCR activation at subcellular resolution in cell cultures to observe activation at the plasma membrane, Golgi apparatus and endosomes.^23^

G‐protein‐based BRET sensors have been developed for several GPCRs, including the β2‐adrenergic receptor (β2AR), α2‐adrenergic receptor (α2AR), vasopressin 2 receptor (V2R), sensor neuron‐specific receptor and thromboxane A2 receptor. In these sensors, a BRET acceptor, GFP10, was fused to G‐proteins and a BRET donor, RLuc, was fused to the GPCR^24^ (Table 1). These sensors can monitor agonist‐induced desensitisation in real time.^24^

**TABLE 1:** Real‐time sensors

Sensor type	NT/NM detection	SNR	Affinity for ligand (EC50/IC50/Kd)	Model	References
FRET based	Acetylcholine	100%	600 nM carbachol	CHO, HEK	^36^, ^43^
	Epinephrine/norepinephrine	32%	12 nM–17 μM	HEK, CHO, PC12	^37^, ^38^, ^42^, ^43^, ^44^, ^57^, ^58^, ^60^, ^62^, ^63^
	Bradykinin	60%	NA	HEK	^45^, ^61^
	Adenosine	16%	NA	HEK	^44^
	Histamine	20%	∼1 μM	HeLa, MEF	^33^
	Lysophosphatidic acid	40%	NA	HeLa	^34^
	Parathyroid hormone	20%	16 nM	HEK	^57^
	Adenine and uridine nucleotides	4%	NA	HEK	^59^
	Glycine (GlyFS)	∼20%	20 μM	HEK, brain tissue	^64^
	Glutamate (FLIPE)	27%	0.6 μM–1 mM	PC12, neuron culture	^65^
BRET based	Epinephrine/norepinephrine	300%	9.1 pM–120 nM isoproterenol	HEK, HeLa, neuron culture and mice	^23^, ^24^, ^32^, ^35^, ^39^, ^40^, ^41^, ^46^, ^53^, ^54^, ^55^, ^56^
	Acetylcholine	450%	0.77 μM carbachol ∼1 μM acetylcholine	HEK	^23^, ^32^, ^35^, ^39^, ^40^, ^56^
	Angiotensin II	130%	1–4.2 nM	HEK	^35^, ^50^, ^54^, ^55^, ^56^
	Chemokine ligands 19 and 21	130%	NA	HEK	^56^
	Chemokine ligands 3, 4, 3‐like 1	∼18%	NA	HEK	^39^, ^55^
	C‐X‐C motif chemokine 12	∼38%	NA	HEK	^39^
	Calcitonin gene‐related peptide	∼20%	NA	HEK	^39^
	Prostaglandin E2	∼55%	NA	HEK	^39^
	Vasoactive intestinal peptide	∼25%	NA	HEK	^39^
	Secretin	∼17%	NA	HEK	^39^
	Glucagon‐like peptide‐1 and glucagon	120%	NA	HEK	^56^
	Platelet‐activating factor	3.6%	NA	HEK	^55^
	Serotonin	130%	NA	HEK	^56^
	Endogenous opioids	2.5%	0.09 nM salvinorin A 5.9–580 nM DAMGO	HEK	^25^, ^39^, ^40^, ^41^, ^55^
	Endocannabinoids	NA	49–65 nM WIN 55,212‐2	HEK	^41^
	Neurotensin	NA	34–390 pM	HEK	^41^
	Adenosine	NA	∼0.5 μM	HEK	^23^
	Thyrotropin‐releasing hormone	NA	8.8 nM	HEK, COS‐1	^47^, ^48^
	Oxytocin	NA	NA	COS‐7	^49^
	Vasopressin	130%	3 nM AVP	HEK and mice	^24^, ^39^, ^50^, ^51^, ^52^, ^55^, ^56^
	Parathyroid hormone	6.8%	10 nM	HEK	^54^
	Dopamine	NA	∼0.1 μM	HEK	^23^, ^39^, ^40^
	Thromboxane	50%	9.6 nM U46619	HEK	^24^, ^35^
	Sensory neuron‐specific receptor	NA	NA	HEK	^24^
	Endothelin‐1	NA	∼1 nM	HEK	^23^
	Bradykinin	NA	NA	HEK	^40^
	Melanocortin	NA	NA	HEK	^50^
SnFRs	Glutamate	∼25	0.6–600 μM	HEK, neuron culture, *Caenorhabditis elegans*, zebrafish, mice, ferrets	^66^, ^67^, ^68^, ^69^
	GABA	∼1.7	30–110 μM	HEK, neuron culture, mice and zebrafish	^70^
	Acetylcholine	14	0.4–35 μM	HEK, neuron culture, *C. elegans*, fly, zebrafish, mouse	^71^, ^73^
	Serotonin	17	390 μM	HEK, neuron culture, mice	^72^
GRAB	Dopamine	3.4	4–130 nM	HEK, neuron culture, fly, zebrafish, mice	^77^, ^89^
	Epinephrine/norepinephrine	2.3	83–930 nM	HEK, neuron culture, zebrafish, mice	^81^
	Serotonin	2.8	14 nM	HEK, neuron culture, flies, mice	^79^
	Endocannabinoids	9.5	9 μM 2‐AG 0.8 μM AEA	HEK, neuron culture, mice	^83^
	Adenosine	∼2.0	60 nM–3.6 μM	HEK, neuron culture, mice	^84^, ^85^
	Acetylcholine	2.8	0.7 μM	HEK, neuron culture, flies, mice	^86^, ^87^
Lights	Dopamine	9.3	4.1 nM–1.6 μM	HEK, neuron culture, mice, rats	^76^, ^90^
	Dynorphin	0.6	NA	HEK, mice	^76^, ^78^
	Serotonin	0.8	26 nM	HEK, neuron culture, mice	^80^
	Epinephrine/norepinephrine	2.1	760 nM	HEK, neuron culture, mice	^76^, ^82^

Abbreviations: ∼Estimated values, because raw values were not available in the publication. All values were rounded to 1–2 significant digits. For all signal‐to‐noise ratios (SNR), the highest values were taken from the publications referenced. For the EC50/Kd values, a range of values were recorded from the publications referenced. EC50/Kd values for different versions of a sensor are included in the range. For those tested in multiple cell types, the SNR and EC50/Kd range are chosen for the cell type that gave the best SNR and lowest EC50/Kd. For fluorescence resonance energy transfer (FRET) and bioluminescence resonance energy transfer (BRET) sensors, the agonist‐induced percent change in BRET/FRET efficiency were recorded as either the agonist‐induced change of fluorescence over the original fluorescence or the signal in the presence of agonist over the background signal without agonist. The SNR of the intermolecular BRET sensors were not recorded in the table because the majority of these sensors were reported to have ‘0’ BRET without agonist, making it impossible to calculate a percent change. BRET, bioluminescence resonance energy transfer; CHO, Chinese hamster ovary cells; FRET, fluorescence resonance energy transfer; GRAB, G‐protein‐coupled receptor (GPCR) activation‐based; HEK, human embryonic kidney cells; MEF, mouse embryonic fibroblast; NT/NM, neurotransmitter/neuromodulator; SnFR, sensing fluorescent reporter; SNR, signal‐to‐noise ratio.

Conformation‐specific nanobodies engineered to bind to active GPCRs have been used as Gα‐mimics and are advantageous in their easy expression in cell culture and high affinities for active GPCRs. These nanobodies have been used in BRET‐based sensors, where one BRET pair is attached to the nanobody and the other is attached to the corresponding GPCR to give an increase in BRET signal upon GPCR activation^25^ (Figure 3B). Alternatively, a nanobody that binds to the inactive GPCR, such as Nb6 for the kappa opioid receptor (KOR), can see a decrease in BRET signal upon GPCR activation.^25^ Although this tool has not been used in any in vivo applications, it was able to observe the different conformational states of KOR induced by different ligands.^25^ A disadvantage of nanobody‐based BRET sensors is that only a few GPCRs have conformation‐specific nanobody binders. These nanobody–GPCR pairs include Nb39 and Nb6 for opioid receptors (ORs),^26^, ^27^ Nb80 for β2AR,^28^ Nb.AT110 for angiotensin receptors^29^ and Nb9‐8 for the M2‐muscarinic receptors^30^ to name a few.

For GPCRs without conformation‐specific nanobodies, miniG proteins can be used as G‐protein mimics. MiniG proteins are derived from the Gα subunit of the G‐protein complex, but with the membrane anchor and Gβγ binding surface removed, thereby allowing easier protein expression than the wild‐type Gα‐protein.^31^ MiniG‐based BRET sensors have been developed by fusing the miniG protein to the BRET acceptor, such as Venus, and fusing the donor, such as Rluc or NLuc, to the GPCR (Figure 3B). In the presence of agonist, miniG can bind to the GPCR and cause an increase in BRET signal. MiniG‐based BRET sensors are very versatile, having been designed for several Gα‐protein types: Gα_i/o_, Gαs, Gα_12/13_ and Gα_q/11_.^23^ Consequently, miniG protein‐based BRET sensors can be used for a wide variety of GPCRs in cell culture; however, to our knowledge, miniG protein‐based BRET sensors have not been used in vivo.

Fusion of the GPCR to the BRET pair involves genetically altering the GPCR; consequently, these types of sensors cannot detect endogenous GPCR activation. Alternatively, BERKY is a BRET‐based GPCR agonist sensor that does not alter the endogenous GPCR and can, therefore, detect endogenous GPCR agonists (Figure 3C). BERKY is a single protein chain where the BRET donor, NLuc, is bound to a membrane anchoring sequence and the acceptor, YFP, is bound to a synthetic peptide called KB‐1753. The donor and acceptor BRET pairs are separated by an ER/K α‐helix linker. Once the Gα‐protein is activated, KB‐1753 interacts with the guanosine triphosphate‐bound Gα‐protein, bringing YFP to the membrane and increasing BRET. BERKY sensors have also been developed for Gα_q_, Gα_13_, Gβγ and Rho to probe the activation of endogenous GPCRs.^32^ Additionally, BRET and FRET pairs inserted into the Gαβγ heterotrimer have also proven useful in measuring endogenous GPCR activation. The proximity of the Gα and Gβγ subunits changes based on the activation state of the GPCR, thereby changing the energy transfer efficiency and indicating GPCR activation^33^, ^34^, ^35^, ^36^, ^37^, ^38^, ^39^ (Figure 3D). Finally, NanoBRET was engineered by fusing Nluc to GRK3ct, a GPCR kinase, and Venus to Gβγ to observe G‐protein activation.^40^ While the above two types of BRET sensors can monitor GPCR activation without direct fusion to the GPCR, they can result in false positives since the sensor activation is dependent on general GPCR activity and not the specific activity of the GPCR of interest. Consequently, control studies without the expression of the GPCR of interest is needed to ensure the sensor activation observed is due to the GPCR of interest.

The advantage of G‐protein binding and activation‐based BRET sensors is that these sensors have been illustrated for a wide variety of G‐protein subtypes (Gα_i_, Gα_o_, Gα_s_, Gα_q_, Gα_12_ and Gα_13_), enabling the detection of a large number of GPCR agonists.^21^ A disadvantage of BRET sensors is that they have poor sensitivity for some G‐protein subtypes.^21^ However, a higher sensitivity can be achieved by systematic optimisation of the insertion points for the BRET pairs. This systematic optimisation was performed in the BRET biosensors TRUPATH, where 14 BRET‐based GPCR sensors were developed.^41^ Still, individual optimisation for each BRET biosensor is strenuous, making it desirable to have a more universal sensor design platform.

In a similar design to the BRET sensors, FRET sensors have been developed with the fluorescent proteins cyan fluorescent protein (CFP) and YFP, where one FRET pair is attached to the GPCR and the other is attached to the G‐protein or G‐protein mimic. The activation of the GPCR causes YFP to come in proximity to CFP and energy transfer can occur (Figure 3B). This sensor type has been developed for the α_2A_AR,^42^, ^43^ the muscarinic‐M4 receptor,^43^ the A_2A_‐adenosine receptor^44^ and the β1‐adrenergic receptor^44^ (Table 1). Enhanced CFP (ECFP) and enhanced green fluorescent protein (EGFP) were used as FRET pairs for the bradykinin receptor type 2.^45^ FRET has faster kinetics than BRET; however, FRET's need for excitation of the donor fluorescent proteins could lead to cell damage or photobleaching. BRET's use of a chemical substrate, therefore, can be less destructive for the cell.

#### 2.1.2 BRET and FRET sensors based on GPCR–β‐arrestin interaction

BRET sensors have been designed based on the intermolecular interaction between β‐arrestin and the active GPCR's C‐terminus. Rluc as the BRET donor has been fused to the C‐terminus of a GPCR and YFP as the BRET acceptor has been fused to β‐arrestin2 to detect the activation of β2AR,^46^ the thyrotropin releasing hormone receptor^47^, ^48^ and the oxytocin receptor^49^ (Figure 3E). Additionally, RLuc and GFP from *Renilla reniformis* have been attached to a GPCR and the transmembrane domain, respectively, where β‐arrestin‐induced internalisation causes a change in energy transfer efficiency.^50^ This sensor type has been designed for the angiotensin II receptor type 1, melanocortin type 4 receptor, and the V2R and can be used to interrogate receptor activation as well as GPCR recycling events.^50^ The GFP and RLuc BRET pair fused to V2R and β‐arrestin, respectively, was also used to interrogate how palmitoylation of the V2R carboxyl tail affects β‐arrestin recruitment.^51^

A BRET sensor with improved efficiency was designed, in which the V2R is fused to hRLuc/RLuc8 and β‐arrestin2 is fused to a modified form of GFP, called 2GFP.^52^ Cells expressing the above BRET sensor were transplanted into a mouse kidney and a transparent, plastic window was fitted into the skin and body wall adjacent to the kidney. The window enables BRET to be measured in deep tissues.^52^ The same BRET pair was used to design a BRET‐based GPCR sensor by fusing β2AR to Rluc and β‐arrestin2 to 2GFP.^53^ Due to poor light penetration in deep tissues, sufficient BRET signal was only achieved in the testes of mice and not in other tissues.^53^ In future work, a red‐shifted BRET pair can improve BRET detection in deep tissues.

BRET sensors have also been developed to detect the specific conformational change found in β‐arrestin upon recruitment to the GPCR. To design this sensor, RLuc and YFP have been attached to either terminus of β‐arrestin,^54^, ^55^ as well as NLuc and a red‐shifted fluorescent protein^56^ (Figure 3F). Different from the above intermolecular BRET sensors, these BRET sensors detect the intramolecular BRET efficiency change due to arrestin's conformational change. Compared to intermolecular BRET and FRET, intramolecular BRET and FRET have a smaller change in the distance between the BRET/FRET pairs and, therefore, a lower signal dynamic range. However, these intramolecular β‐arrestin‐based BRET sensors can observe the different types of active conformations that β‐arrestin adopts and have been used for a variety of GPCRs, such as the angiotensin 1 receptor, vasopressin receptors 1 and 2, β1 and β2‐adrenergic receptors, muscarinic 1 receptor, chemokine receptor 5, delta opioid receptor (DOR), serotonin receptor, platelet‐activating factor receptor, chemokine receptor type 7 and the glucagon‐like peptide‐1 receptor.^54^, ^55^, ^56^

Similar to the G‐protein‐based FRET design, CFP and YFP have been used as FRET pairs. One FRET pair is attached to the GPCR and the other is attached to β‐arrestin1 or 2 (Figure 3E). These sensors have been applied in cell cultures to determine the time it takes β‐arrestin2 to be recruited to the parathyroid hormone 1 receptor^57^ and β2AR.^58^ They have also been applied to determine the differential recruitment of β‐arrestin1 and 2 to P2Y2R.^59^

Compared to the G‐protein‐based sensors, β‐arrestin‐based BRET and FRET real‐time sensors can generate a stable on‐signal, because the GPCR–β‐arrestin interaction occurs on the order of minutes to tens of minutes, while the GPCR–G‐protein interaction is much more transient, on the order of seconds. Consequently, β‐arrestin‐based BRET and FRET real‐time sensors are also limited by their off kinetics. Additionally, not all GPCRs strongly recruit β‐arrestin. Therefore, G‐protein‐ and β‐arrestin‐based BRET and FRET sensors should be chosen depending on their different applications.

#### 2.1.3 BRET and FRET sensors based on the conformational changes in the GPCR

G‐protein‐based BRET sensors require knowledge of the specific G‐protein type that binds to the GPCR which is not always known. β‐Arrestin‐based BRET sensors need to use GPCRs that couple strongly to β‐arrestin which, as stated previously, is not true for all GPCRs. To address these limitations in sensor designs, BRET and FRET sensors have been developed that utilise the conformational change of the GPCR upon activation rather than utilising the intermolecular interactions of the G‐proteins and β‐arrestin with the GPCR. Theoretically, these types of sensors should be more universal given that all GPCRs undergo a conformational change upon activation. CFP can be inserted into the third intracellular loop of the GPCR where the major conformational change takes place upon GPCR activation, and YFP can be fused to the C‐terminus of the GPCR, or the other way around (Figure 3G). This strategy has been used for the parathyroid hormone receptor 1 and α_2A_AR to observe the different conformational changes of GPCRs induced by different types of ligands.^57^, ^60^ These sensors have been developed for other types of GPCRs as well^61^, ^62^, ^63^ (Table 1). These intramolecular sensors based on the conformational changes in the GPCR have a lower signal dynamic range than the intermolecular BRET and FRET sensors.

#### 2.1.4 BRET and FRET for detecting the conformational change of binding proteins

**FIGURE 4:**
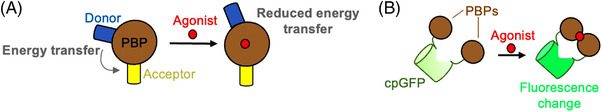
Periplasmic binding protein (PBP)‐based tools. (A) Resonance energy transfer pairs are attached to PBPs. Ligand binding induces a conformational change in the PBP, which results in a change in energy transfer efficiency. (B) PBPs are attached to either terminus of circularly permuted green fluorescent protein (cpGFP), where ligand binding to the PBP causes a conformational change in cpGFP, resulting in a fluorescence change.

Although the majority of FRET/BRET sensors for GPCR agonists involve GPCRs or G‐proteins in their design, the glycine FRET sensor (GlyFS) and the glutamate sensor (FLIPE) use soluble protein domains that can bind to glycine and glutamate in their sensor designs. In GlyFS, two FRET pairs, ECFP and Venus, were both attached to a rationally designed soluble glycine binding protein.^64^ Glycine binding to the sensor reduces the FRET between the two fluorescent proteins (Figure 4A). GlyFs was used in acute hippocampal slices to reveal changes of extracellular glycine concentrations during development, enrichment of glycine outside of the synapse, and the increase of extracellular glycine from neuroplasticity.^64^ The glutamate sensor, FLIPE, has a similar design to GlyFs, where ECFP and Venus were inserted into a soluble glutamate binding protein, ybeJ.^65^ FLIPE was used to show that glutamate uptake in the cytosol of PC12 cells has a minimal effect on overall cytosolic glutamate levels.^65^

#### 2.1.5 Summary of advantages and limitations of BRET‐ and FRET‐based GPCR sensors

BRET‐ and FRET‐based sensors have been designed for a wide variety of GPCRs, mainly based on the activated GPCR's interaction with G‐proteins or β‐arrestin or the conformational change of the GPCR to indicate GPCR activation. BRET and FRET designs are highly versatile and can be generally applied to most GPCRs by using different types of Gα‐proteins or β‐arrestin. However, BRET is limited by the low signal intensity of luminescence which requires longer BRET acquisition time. FRET is limited in its signal‐to‐noise ratio (SNR), which is most commonly <100%, due to potential spectral overlap or non‐proximity‐based energy transfer. Here, we define SNR for real‐time sensors as either the agonist‐induced change of fluorescence over the initial fluorescence or the signal over background ratio. Additionally, these sensors take extensive engineering to determine the correct placement of the donor and acceptor, so the specific conformation‐induced change of the distance of the FRET donor–acceptor pair can be maximal. Lastly, BRET and FRET require the use of two fluorescence channels, limiting the use of multiple fluorescence markers and sensors.

To overcome these limitations, single fluorescent‐protein‐based systems have been developed by using cpGFP. Circular permutation involves the fusion of the original N‐ and C‐terminus of a protein and the creation of a new N‐ and C‐terminus in a different position on the protein. cpGFP was engineered to have its new N‐ and C‐terminus near the fluorophore pocket, thereby making the fluorophore more exposed to the solvent environment. Environmental changes around the cpGFP fluorophore can cause a change in the fluorescence emission intensity. This resulting change in fluorescence is instantaneous and reversible, making cpGFP a good tool for real‐time detection of neurotransmitters and neuromodulators. cpGFP as a real‐time sensor has been used in two ways: (1) cpGFP inserted into periplasmic binding proteins; and (2) cpGFP inserted into the third intracellular loop of a GPCR.

### 2.2 Single‐colour fluorescent sensors based on cpGFP insertion into periplasmic binding proteins

Fluorescent sensors for several neuromodulators and neurotransmitters have been engineered using periplasmic binding proteins, receptors found in bacteria that undergo a conformational change in the presence of a variety of small molecules. Specifically, these are Venus‐fly trap‐like proteins that close in the presence of their binding ligand. cpGFP was inserted into periplasmic binding proteins to create a series of neurotransmitter and neuromodulator sensors, named sensing fluorescent reporters (SnFRs). For the SnFR design, ligand binding causes a conformational change in the periplasmic binding proteins which causes a change in the fluorophore environment of cpGFP, resulting in a fluorescence change (Figure 4B). SnFRs have been developed to detect several GPCR ligands, including glutamate, GABA, acetylcholine and serotonin, with SNR from 1 to 25 (Table 1). The glutamate version of SnFR, called iGluSnFR, has been used to detect glutamate in *Caenorhabditis elegans*, zebrafish, mice and ferrets.^66^ The original iGluSnFR used circular permuted enhanced GFP (cpEGFP) but new versions have since been developed with circularly permuted super folder GFP (SF‐iGluSnFR) which has higher expression level and fluorescent signals.^67^ Additionally, different coloured variants and variants with a variety of affinities for glutamate have been developed.^67^ More recently, iGluSnFR3 was developed through directed evolution and has a higher SNR, dynamic range, expression and photostability than SF‐iGluSnFR.^68^ Finally, faster versions of iGluSnFR have been developed to resolve individual glutamate release events in rat hippocampal slices.^69^ The SnFR engineering technique has also been used to engineer a sensor for an important inhibitory neurotransmitter, GABA. iGABASnFR has been used in vivo to track the concentration of GABA in the mitochondria in zebrafish models and track the GABA signalling dynamics during interictal spikes and seizures in mice.^70^ Recently, iAChSnFR was developed to detect acetylcholine release in mice, zebrafish, flies and *C. elegans*.^71^

Naturally occurring periplasmic binding proteins do not exist for the majority of neurotransmitters and neuromodulators, limiting the generalizability of the SnFR design; however, engineering of the binding pocket of existing periplasmic binding proteins could lead to the development of new sensors. Using machine learning, researchers re‐engineered the binding pocket of iAChSnFR's periplasmic binding protein to bind to serotonin, generating iSeroSnFR. iSeroSnFR was used to detect serotonin release in mice during different behavioural assays, such as fear conditioning, social interaction and sleep/wake transitions.^72^ Further, site‐saturated mutagenesis of a periplasmic binding protein was performed to select for a variant that can bind specifically to nicotinic acetylcholine receptor agonists, thereby creating iNicSnFR. iNicSnFR was used to determine the rate at which different nicotinic receptor agonists leave the endoplasmic reticulum.^73^ The iNicSnFR was subsequently mutated using site‐saturated mutagenesis and rational design to select for variants that specifically bind to S‐methadone and not cholinergic ligands; this sensor is called iS‐methadoneSnFR.^74^

To further illustrate the versatility of the SnFR design platform, a new set of SnFRs were developed recently for a series of smoking‐cessation drugs: dianicline, cytisine, 10‐fluorocytisine and 9‐bromo‐10‐ethylcytisine. These sensors are called iDrugSnFRs and have been used to determine the differing rates of membrane crossing of smoking‐cessation drugs, a property that affects the drug's pharmokinetics.^75^

The application of the SnFR sensors has been demonstrated in a variety of model organisms to interrogate the neurobiology underlying various neurotransmitter and neuromodulator‐mediated signalling events. The wide applicability of SnFRs for in vivo experiments illustrates the usefulness and robustness of the sensor design.

### 2.3 Single‐colour fluorescent sensors based on cpGFP insertion into the third intracellular loop of GPCRs

**FIGURE 5:**
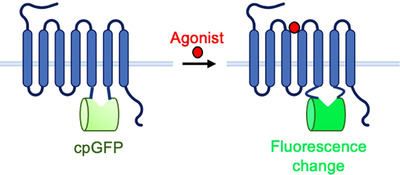
Circularly permuted green fluorescent protein (cpGFP) inserted into the third intracellular loop of a G‐protein‐coupled receptor (GPCR). Upon agonist‐induced GPCR activation, the third intracellular loop of the GPCR undergoes a conformational change, changing the fluorophore environment of cpGFP and resulting in a fluorescence change.

GPCR activation‐based (GRAB) and light sensors were developed by inserting cpGFP into the third intracellular loop of a GPCR. Upon agonist binding, the third intracellular loop undergoes a conformational change that changes the fluorophore environment of cpGFP, resulting in a fluorescence change (Figure 5). These sensors have been developed to detect dopamine,^76^, ^77^ endogenous opioid peptides,^76^, ^78^ serotonin,^79^, ^80^ noradrenaline,^76^, ^81^, ^82^ endocannabinoids,^83^ adenosine^84^, ^85^ and acetylcholine^86^, ^87^ with most SNRs ranging between 2 and 10 (Table 1). For some versions of these sensors, portions of the third intracellular loop have been truncated to allow a larger fluorescence change. Different linkers connecting cpGFP to the third intracellular loop have also been screened to change the sensitivity and SNR of the sensors. Both rational design and site‐saturated mutagenesis have been used to engineer these sensors and improve their SNR and overall brightness.^88^ In addition, red‐shifted versions have been developed for both GRAB and light sensors to allow for multiplexed imaging of different neurotransmitters and neuromodulators at a single time.^89^, ^90^ For a more comprehensive overview of the engineering used to design the GRAB/light sensors, see reference.^88^

GRAB sensors have been used in a variety of in vivo models, including flies, zebrafish and mice.^77^ Light sensors have been illustrated in vivo in mice and rats.^76^ These sensors were used in animal models to reveal important information about neurotransmitter and neuromodulator‐mediated neurobiology. To highlight a few applications, the GRAB_Ado_ sensor was used to study sleep homeostasis and found that adenosine is at high concentration during rapid eye movement (REM) sleep, but low outside of the REM sleep cycle.^84^ Using GRAB_Ado_, researchers also found that the activation of glutamatergic neurons resulted in an increase of adenosine. Further, dLight helped researchers find that heroin activates dopaminergic neurons located in the medial area of the VTA,^91^ dopamine is involved in relapse and alcohol‐seeking behaviours,^92^ and that the pattern of morphine exposure affects dopamine release in the nucleus accumbens.^93^ GRAB_DA_ was used to show that alcohol, but not fat, significantly increased dopamine in the nucleus accumbens and that fat, but not alcohol, increased dopamine in the dorsal striatum.^94^

### 2.4 Translocation assays

Rather than genetically altering the GPCR, translocation assays can be performed to indicate GPCR activation. When a GPCR is activated, GPCR‐interacting proteins can translocate from the cytoplasm to the cell membrane or from the membrane to the cytoplasm. Consequently, these interacting proteins, such as β‐arrestin,^95^, ^96^, ^97^ protein kinase C^95^ and G‐proteins/mimics^98^, ^99^ have been tagged with fluorescent proteins and their agonist‐induced translocation has been detected with high‐magnification imaging. Total internal reflection fluorescence microscopy has also been used to analyse the fluorescence ∼100 nm from the cell surface, allowing measurements of fluorescence on the membrane only.^98^ A major limitation of translocation assays is their low SNR.

### 2.5 Detection of second messengers

The activation of a GPCR can increase or decrease the concentration of second messengers, such as cAMP, IP_3_, Ca^2+^ and diacylglycerol. Consequently, sensors that can detect the concentration of second messengers can be used to detect GPCR activation by an agonist. These sensors can be generalizable to many GPCRs, because the activation of most GPCRs will cause a change in the concentration of at least one of the second messengers previously mentioned. However, second messaging events can also be caused by non GPCR‐mediated signalling, potentially giving false positive signals. It is, therefore, better to use GPCR agonist sensors that detect more upstream signals, such as conformational changes in the GPCR or β‐arrestin/G‐protein binding, when available.

**FIGURE 6:**
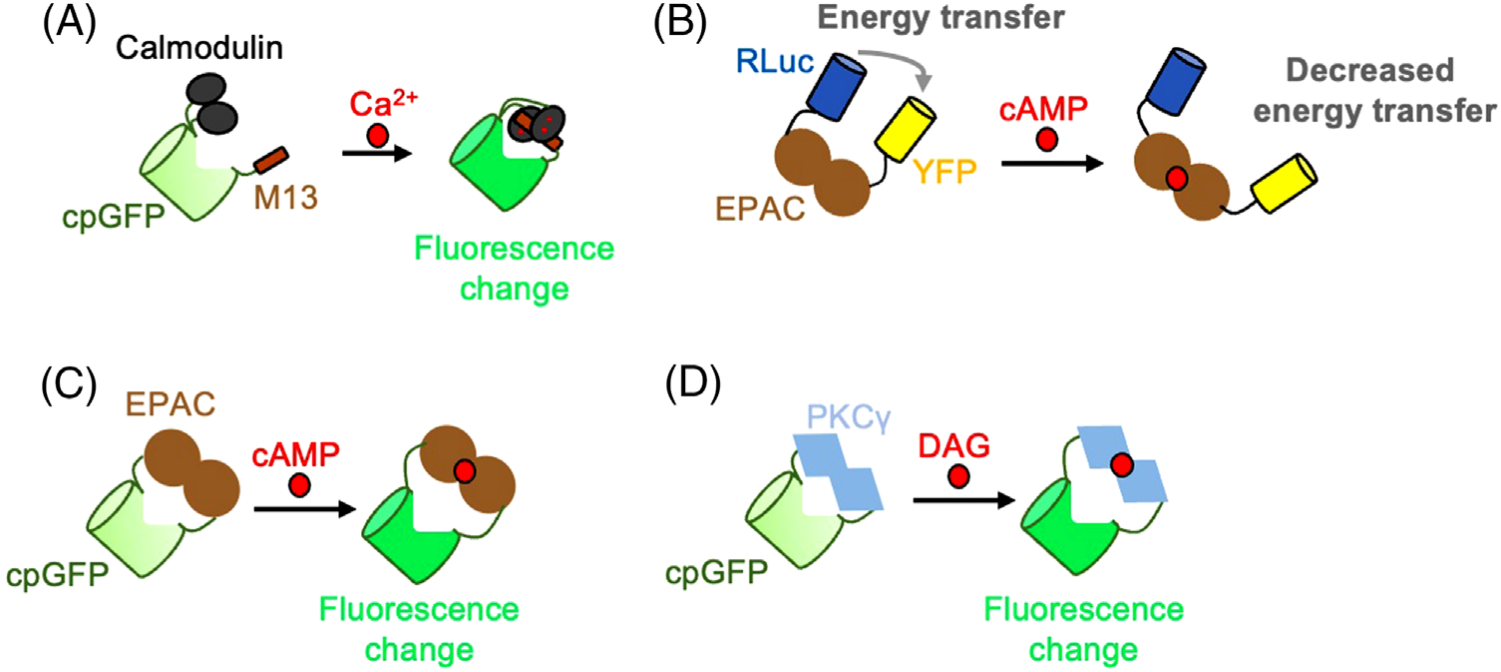
Sensors that detect secondary messengers. (A) Calcium‐sensing proteins, calmodulin and M13, are attached to either terminus of circularly permuted green fluorescent protein (cpGFP). In the presence of calcium, calmodulin and M13 interact, resulting in a change in the fluorophore environment of cpGFP and a fluorescence increase. (B) Schematic of CAMYEL. Bioluminescence resonance energy transfer (BRET) pairs RLuc and yellow fluorescent protein (YFP) are tethered to EPAC. cAMP binding causes a conformational change in EPAC, where the energy transfer efficiency is decreased. (C) EPAC was attached to either terminus of cpGFP. cAMP‐induced EPAC conformational change results in a change in cpGFP fluorescence. (D) PKCγ, which only binds to diacylglycerol (DAG), is tethered to either terminus of cpGFP. DAG induces a conformational change in PKCγ, changing the cpGFP fluorescence.

One of the most widely used second messenger sensors, GCaMP, was first developed in 2001 to detect intracellular calcium changes in living cells.^100^ GCaMP involves the fusion of a calcium‐dependent protein binding pair, M13 and calmodulin, to either terminus of cpGFP. In the presence of calcium, these proteins bind, changing the fluorophore environment of cpGFP and resulting in a fluorescence change (Figure 6A). After 20 years of dedicated work in sensor optimisation, improved versions of GCaMP have been published that have better SNR, heightened brightness, faster kinetics and heightened calcium sensitivity than the original version.^101^, ^102^, ^103^, ^104^ GCaMP has been used in a variety of model organisms in vivo, such as *C. elegans*,^102^, ^104^ flies,^101^, ^102^, ^103^, ^104^ mice^101^, ^102^, ^103^, ^104^ and zebrafish.^101^, ^102^ Further, GCaMP has been used in vivo to detect calcium spiking in axons, illustrating its subcellular resolution.^105^ The impact of GCaMP on the field of neuroscience cannot be understated. Because this review focuses on neuromodulators and not calcium sensors, a more extensive review of GCaMP and its different optimised versions, as well as other genetically encoded calcium sensors, can be found in references.^106^, ^107^

A FRET sensor used to detect cAMP, called CAMYEL, was developed by inserting a protein that interacts with cAMP, EPAC, in between two BRET pairs, YFP and RLuc.^108^ Upon the binding of cAMP to CAMYEL, there is a quantifiable decrease in BRET signal (Figure 6B). This tool was used to discover that sphingosine 1‐phosphate can increase the amount of intracellular cAMP that is stimulated by isoproterenol and prostaglandin E_2_.^108^ In addition to CAMYEL, many other FRET sensors for cAMP detection have been developed and are summarised in reference.^109^ EPAC was also inserted into cpGFP, where cAMP binding to cpGFP causes a change in cpGFP fluorescence intensity^110^ (Figure 6C).

A fluorescent sensor for diacylglycerol was developed by fusing cpGFP to an isoform of protein kinase II that only responds to diacylglycerol^111^ (Figure 6D). This diacylglycerol sensor was, then, improved by replacing cpGFP with the brighter mNeonGreen fluorescent protein.^110^ Additionally, FRET was used to develop a sensor for protein kinase A activity, an important protein in downstream GPCR signal transduction.^112^

## 3 GPCR agonist integration sensors

Integration‐based GPCR agonist sensors integrate the GPCR agonist signal into a permanent and measurable readout for further analysis post mortem. Integration sensors have complementary strengths to real‐time sensors by enabling the examination of the localisation of GPCR agonists across a large area of interest or the whole brain to interrogate a GPCR's effect on neuronal circuitry globally. Additionally, through permanent neuronal labelling, integrators allow further interrogation of the neuronal circuits of interest. Integration‐based GPCR agonist sensors have been engineered for a wide variety of GPCRs and can be divided into three main categories based on their signal output: (1) transcriptional activation; (2) cpGFP fluorophore formation; and (3) split protein complementation sensors.

### 3.1 Transcriptional activation‐based GPCR agonist integration sensors

**FIGURE 7:**
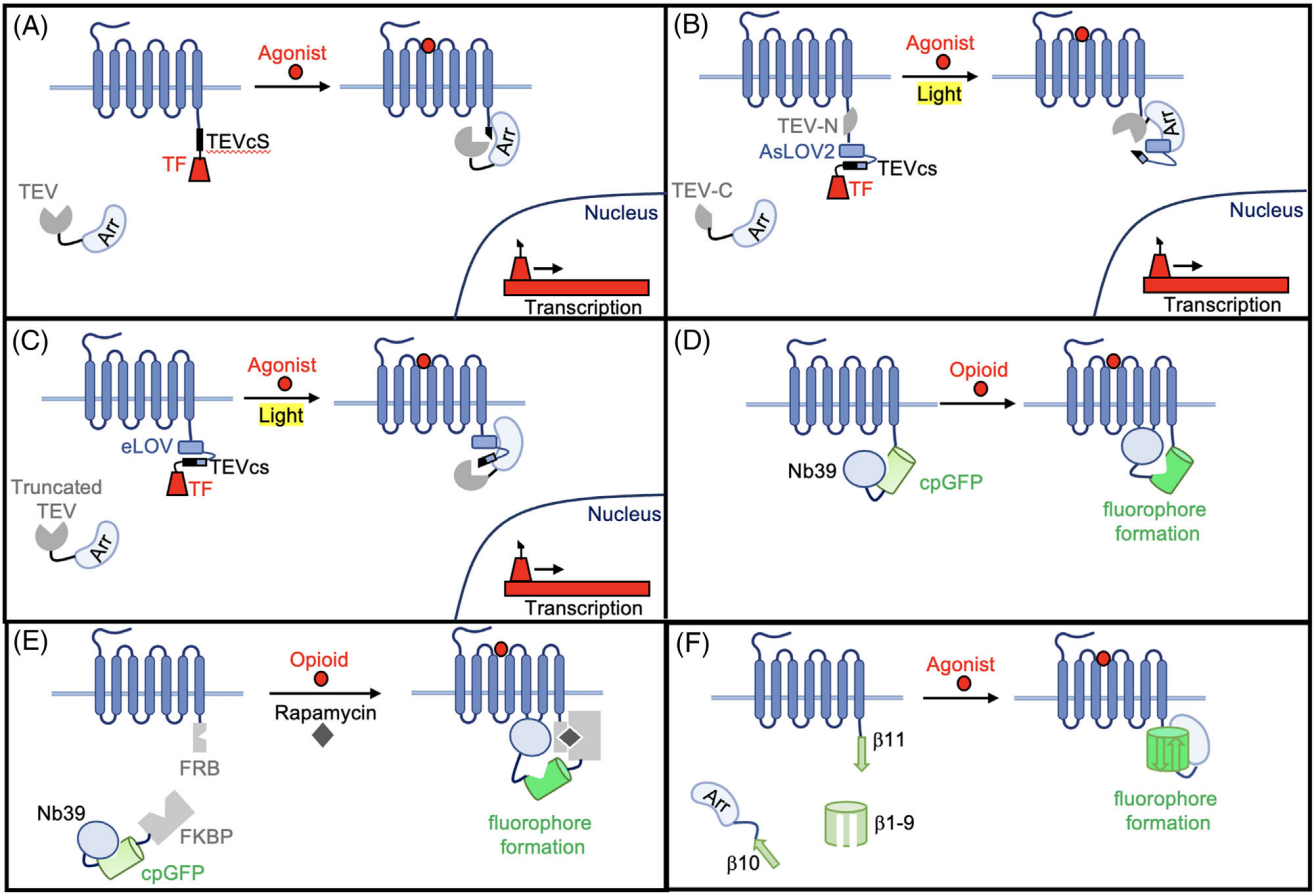
Integrators. (A) Schematic of Tango. The C‐terminus of the G‐protein‐coupled receptor (GPCR) is fused with a tobacco etch virus protease cleavage site (TEVcs) and transcription factor (TF). β‐Arrestin is tethered to a TEV protease. Agonist binding to the GPCR recruits β‐arrestin, where the TEV protease can then cut at the TEVcs, releasing the TF so it can translocate to the nucleus and activate a reporter gene. (B) Schematic of *i*Tango2. Same basic mechanism as Tango, except the TEV protease is split into two components (TEV‐N and TEV‐C) that are fused to β‐arrestin and the GPCR. Additionally, the light oxygen voltage sensing (AsLOV2) domain cages the TEVcs. Agonist recruits β‐arrestin fused split TEV to the GPCR, where the split protease components can reassociate and light uncages TEVcs, allowing the protease to cut and release the TF. (C) Schematic of SPARK. Same basic mechanism as *i*Tango2, except the TEV protease is not split, a truncated protease is used instead. Additionally, evolved LOV (eLOV) is used instead of AsLOV2. (D) Schematic of SPOTIT. Circularly permuted green fluorescent protein (cpGFP) and Nb39 are tethered to the C‐terminus of the GPCR. Nb39 inhibits cpGFP fluorophore formation. Agonist activates the opioid receptor (OR), recruiting Nb39 to the third intracellular loop, releasing cpGFP and allowing the fluorophore to form. (E) Schematic of SPOTon. Same basic mechanism of SPOTIT, except cpGFP‐Nb39 is fused to FK506 binding protein (FKBP) and the OR is fused to FKBP‐rapamycin binding domain (FRB). Rapamycin induces heterodimerisation of FKBP and FRB, bringing cpGFP‐Nb39 to the OR. Opioid activates the OR, recruiting Nb39 to the third intracellular loop and allowing the cpGFP fluorophore to mature. (F) Schematic of Trio. GFP is split into three component: β1‐9, β10 and β11. β10 is attached to β‐arrestin, β11 is attached to the GPCR and β1‐9 is expressed in the cytosol. Agonist‐induced GPCR activation recruits β‐arrestin to the C‐terminus of the GPCR, allowing the three split components of GFP to re‐associate and a fluorescence increase.

A transcriptional activation‐based GPCR sensor uses unique transcription factors and promoters that are orthogonal with the cellular system to activate a reporter gene upon agonist binding. The reporter genes commonly code for fluorescent proteins, so that activation of the sensors by GPCR agonists will fluorescently label the cells. Transcription‐based GPCR sensors were first developed in 2008, in which the C‐terminus of the V2R was fused to the tTA transcription factor via the N1a tobacco etch virus (TEV) protease cleavage site (TEVcs), and β‐arrestin2 was fused to TEV protease^113^ (Figure 7A). In the presence of the V2R agonist, the β‐arrestin2 fused to TEV protease will bind to the C‐terminus of V2R, bringing the protease in proximity to TEVcs for cleavage to take place, releasing tTA. No longer tethered to the membrane, tTA can then translocate to the nucleus and activate a tTA‐dependent reporter gene. This sensor design, called Tango, was developed for 89 other GPCRs, illustrating the robustness of the technique.^113^ It is important to note, however, that the V2R's C‐terminal tail needs to be added to the other GPCRs to enhance β‐arrestin2 recruitment and consequently increase the SNR. Here, we define SNR of integrators as the signal over background ratio.

To apply Tango to the majority of the druggable GPCRs within the human genome, PRESTO‐Tango was developed.^114^ PRESTO‐Tango uses a ‘modular design strategy’ to efficiently design Tango‐based GPCR sensors for over 300 GPCRs. The researchers, then, demonstrated these 300+ Tango‐based sensors can be used to test compounds against nearly the entire druggable human GPCR genome. They tested two compounds against 133 GPCR‐Tango targets and showed that one compound, lysergic acid diethylamide, has activities against GPCRs that were not previously known.^114^ Additionally, they screened 91 GPCR‐Tango targets against FDA‐approved drugs and found that the diabetes drug, nateglinide, has activity against MRGPRX4, a receptor thought to be involved in pain and itch.^114^

The PRESTO‐Tango system represents one of the most widely applied high‐throughput screening platform for GPCR ligands. However, its application in detecting GPCR ligands has so far been limited to cell cultures. In the PRESTO‐Tango system, the TEV protease has basal cleavage activity that could accumulate over time in animal models, reducing the SNR. Additionally, Tango activation requires hours of GPCR agonist incubation due to the low catalytic efficiency of the TEV protease. To overcome these limitations, a light‐activated Tango was developed in 2017 called inducible Tango (*i*Tango2).^115^

Because integrators can start accumulating background signal immediately after the sensor is expressed, *i*Tango2's use of a light gating reduces the overall background of the system and allows signal to accumulate only in the light window. Additionally, light can be used as a temporal gating to record signalling events during a specific behaviour. The *i*Tango2 design differs from Tango in two main ways (Figure 7B). First, the TEV protease is split into two halves, where one is tethered to β‐arrestin2 and the other is attached to the C‐terminus of the GPCR. Second, an engineered lightsensing protein based on *Avena sativa* phototropin 1 light‐oxygen‐voltage 2 domains (AsLOV2) is added to cage the TEVcs in the dark state. In light, the Jα helix of the AsLOV2 protein undergoes a conformational change, uncaging the TEVcs to be accessible to the TEV protease. Agonist activation recruits β‐arrestin2, bringing split TEV protease half TEV‐C to the GPCR‐TEV‐N fusion protein, and allowing the split TEV protease to reconstitute. Therefore, only in the presence of both light and agonist activation will the split TEV protease be reconstituted and can cleave the light‐uncaged TEVcs, releasing tTA. *i*Tango2 showed a SNR 20‐fold higher than the original Tango system.^115^ *i*Tango2 has been developed for DRD2,^115^ cannabinoid receptor type 1,^115^ serotonin receptor 1A,^115^ neuropeptide Y receptor type 1^115^ and the oxytocin receptor^116^ (Table 2). Additionally, *i*Tango2 has been used to label dopamine‐sensitive neuronal populations during reward‐based learning in mice^115^ and was used to detect the change of CCR5 activity after learning in mice.^117^

**TABLE 2:** Integrator sensors

Sensor type	NT/NM detection	SNR	EC50	Model	References
TANGO	Has been developed for over 300 GPCRs	1.3–180	NA	HEK, neuron culture, mouse	^113^, ^114^
*i*TANGO	Dopamine	8.9	∼50 nM	HEK, neuron culture, mice	^115^, ^117^
	Cannabinoid	∼3.6	NA	Neuron culture	^115^
	Serotonin	∼4.5	NA	Neuron culture	^115^
	Neuropeptide Y	∼6.3	NA	Neuron culture	^115^
	Oxytocin	6.6	∼2.5 μM	HEK, neuron culture, mice	^116^
SPARK	Epinephrine/norepinephrine	24	52 nM iso	HEK	^118^, ^122^
	Motilin	18	NA	HEK	^118^
	Dopamine	19	NA	HEK	^118^
	Bombesin	37	NA	HEK	^118^
	Vasopressin	4.2	NA	HEK	^118^
	Angiotensin II	1.4	NA	HEK	^118^
SPOTIT	Opioid peptides	MOR: 13 KOR: 38 DOR: 2.7	15 nM fentanyl 0.77 μM salvinorin A	HEK and neuron culture	^123^, ^124^, ^125^
Trio	Epinephrine/norepinephrine	NA	NA	HEK	^127^
	Neurokinin	NA	NA	HEK	^127^
	Opioid peptides	NA	NA	HEK	^127^

*Note*: ∼Estimated values, because raw values were not available in the publication. All values were rounded to 1–2 significant digits. For all signal‐to‐noise ratio (SNR), the highest values were taken from the publications referenced. For the EC50/Kd values, a range of values were recorded from the publications referenced. EC50/Kd values for different versions of a sensor are included in the range. For those tested in multiple cell types, the SNR and EC50/Kd range are chosen for the cell type that gave the best SNR and lowest EC50/Kd. For the integrator sensors, the SNR was calculated as the signal in the presence of agonist over the background signal without agonist. For the light‐gated integrators, the agonist‐dependent SNR in the presence of light was recorded.

Abbreviations: DOR, delta opioid receptor; GPCRs, G‐protein‐coupled receptors; HEK, human embryonic kidney cells; KOR, kappa opioid receptor; MOR, mu opioid receptor; NT/NM, neurotransmitter/neuromodulator; SNR, signal‐to‐noise ratio.

Similar to *i*Tango2, SPARK is a light‐controlled, transcription‐based GPCR agonist sensor that utilises TEV protease cleavage to release a unique transcription factor that is tethered to the GPCR and can activate reporter gene expression^118^, ^119^, ^120^ (Figure 7C). SPARK differs from *i*Tango2 in two main ways. First, it does not use split TEV protease and instead uses a C‐terminal truncated TEV protease that has low affinity for the TEVcs. This allows protease cleavage only to occur when the TEV protease is brought to proximity to the TEVcs. Second, the TEVcs is caged by an evolved version of the AsLOV2 domain (eLOV). eLOV was evolved using directed evolution and was shown to have a 10× better light‐to‐dark signal ratio than the original AsLOV2.^121^ In the initial testing of the SPARK design for detecting GPCR agonists, β2AR was fused to the eLOV domain‐caged TEVcs and a GAL4 transcription factor and β‐arrestin was fused to the truncated TEV protease. To illustrate the generalizability of SPARK in GPCR agonist detection, SPARK was tested with eight different GPCRs. Six of these GPCRs had robust light‐ and ligand‐dependent gene activation with five having ligand‐dependent SNR above 15^118^ (Table 2).

One main advantage of the transcriptional system is the versatility of the signal output. To illustrate this, researchers used β2AR‐SPARK to drive luciferase expression, illustrating how SPARK can be used for high‐throughput screening assays that need an easily quantifiable readout.^118^ SPARK and *i*Tango were compared using the β2AR versions of both tools and transfection in HEK293T cells. SPARK was found to have a 16.4‐fold higher ligand‐dependent SNR^118^ and a higher sensitivity than *i*Tango2.^118^

Recently, SPARK2 was developed using an evolved TEV protease, uTEV1Δ, with improved catalytic efficiency.^122^ SPARK's activation efficiency is limited to the number of cleavage events performed by the TEV protease. Due to the relatively slow catalytic rate of the TEV protease, SPARK requires 10–15 min of light and agonist stimulation to see a sufficient signal, limiting its application in situations where the GPCR agonist‐induced protein–protein interaction (PPI) occurs on the order of seconds to a few minutes. SPARK2 uses uTEV1Δ which has a faster cleavage rate than the original TEV protease, and consequently only 1 min of light and agonist stimulation is needed to see a robust signal for β2AR‐SPARK2.^122^

### 3.2 cpGFP fluorophore formation‐based GPCR agonist integration sensors

SPARK and TANGO are multi‐component systems, where the SNR is highly protein expression level‐dependent and multiple viruses need to be delivered in animal model studies. Single protein chain‐based integration systems could overcome some of the limitations of the multi‐component systems for interrogating GPCR signalling in a whole brain at cellular resolution. Recently, cpGFP‐based sensors with a fluorophore formation‐based mechanism have been developed to detect opioids for the mu opioid receptor (MOR), KOR, and delta opioid receptor (DOR)^123^ (Table 2). Named SPOTIT1, these tools take advantage of the ability of Nb39 to both bind to the active MOR and inhibit cpGFP fluorophore formation, thereby providing a sufficient protein switch for opioid detection. As shown in Figure 7D, in the absence of opioids, Nb39 remains bound to cpGFP and cpGFP fluorophore formation is inhibited. In the presence of opioids, Nb39 dissociates from cpGFP to bind to the active OR. This allows the cpGFP fluorophore to form and, therefore, a subsequent fluorescence increase. SPOTIT1 showed a SNR up to 12.5 in HEK293T cell culture and was shown to be effective in detecting a variety of opioid agonists, such as peptide agonists, synthetic agonists and partial agonists. SPOTIT1 was also shown to be functional in neuronal cell culture, illustrating its potential applicability in animal models. However, SPOTIT1 had reduced brightness in neuronal cell culture.^123^ To improve the brightness of the SPOTIT sensors, SPOTIT2 was developed by adding four amino acids to the N‐terminus of cpGFP to better encapsulate the fluorophore and increase its quantum yield.^124^ In HEK293T cell culture, SPOTIT2 is 11× brighter than SPOTIT1 and 2.7× brighter in neuronal culture. Our lab recently tested SPOTIT2 in mice and a significant difference in signal was detected between the morphine‐ and saline‐treated mice (unpublished data), illustrating SPOTIT2's potential applications in animal models.

As an integration reporter, SPOTIT accumulates opioid signal over time until reaching a saturation point. Similarly, the background signal, which arises from the basal activity of the ORs, could also accumulate over time. To minimise the background signal accumulation, a chemical‐gated version of SPOTIT, called SPOTon, was designed.^125^ In the SPOTon sensor design, SPOTIT is split into two components which are fused to a heterodimerising PPI pair, FK506 binding protein (FKBP) and FKBP‐rapamycin binding domain (FRB); the OR was fused to FRB and cpGFP‐Nb39 was fused to FKBP. In the presence of the small molecule rapamycin, FKBP and FRB heterodimerise, bringing cpGFP‐Nb39 to the OR and creating a functional opioid sensor. Then, the opioid‐activated OR can recruit Nb39, allowing the cpGFP fluorophore to form. This sensor had a comparable opioid‐dependent SNR as the SPOTIT sensors and strong rapamycin‐dependence.^125^ Due to rapamycin's low blood–brain barrier (BBB) penetrability, the engineering of a chemical‐gated SPOTon that is activated with a BBB penetrable molecule would be needed for animal studies. Further, even though SPOTIT has only been demonstrated to detect OR agonists, the SPOTIT design could potentially be extended to other GPCR agonists by using other conformational specific binding nanobodies.

### 3.3 Split fluorescent protein complementation

Termed bimolecular fluorescence complementation (BiFC), split fluorescent protein components have low fluorescence when the split fluorescent protein halves are apart, due to the inability of the fluorophore to form, and a high fluorescence when the components re‐associate. Split fluorescent protein can be designed to be proximity dependent by tuning the binding affinity of the split components, such that the complementation of the components only occurs when they are brought into proximity. These proximity‐dependent split fluorescent protein can then be fused to PPI pairs to detect PPIs (Figure 7F).

Split GFP has been used extensively in BiFC assays to detect GPCR agonists. GFP has a 11‐stranded β‐barrel structure and, due to its very stable structure, can be split in a variety of positions. Tripartite GFP, where GFP is split at β‐strand 10 and 11 to give three fragments: β10, β11 and β1‐9, has better SNR than a bipartite system due to reduced reassociation of the split fragments in the absence of PPI.^126^ Tripartite GFP was used to detect GPCR activation by fusing β11 to the C‐terminus of a GPCR, β10 to β‐arrestin, and expressing β1‐9.^127^ Agonist‐induced GPCR activation recruits β‐arrestin to the GPCR's C‐terminus, allowing all three split fragments to re‐associate and fluorophore formation to occur (Figure 7F). This assay, named Trio, was generated for the protease‐activated receptor 1, β2AR, neurokinin receptor and MOR (Table 2). However, similar to Tango and SPARK, this system is limited to GPCRs that recruit β‐arrestin and is a multiple component system.

## 4 SUMMARY

Tables 1 and 2 list various real‐time sensors and integration sensors for a range of GPCR agonists discussed in this review. When choosing which existing sensor to use, one must consider both the SNR and affinity of the agonist for the sensor. Higher SNRs are generally preferred to provide a more robust and sensitive application in animal models. Sensors with a range of affinities for the agonist need to be evaluated in the specific animal model used for testing to determine which sensor can detect agonist concentrations with the highest dynamic range. While some sensors have been validated in animal models, many sensors have only been tested in cell cultures. For the latter, careful evaluations in animal models are needed before the sensors can be applied to study endogenous agonist release. In some cases, further engineering of the sensor SNR or affinity for ligand might be needed to achieve robust application in animal models.

Generally, real‐time sensors have lower SNRs than integration sensors, partially due to the mechanism of the sensor design and also their transient detection, while integration sensors can accumulate signals over time to provide an end point readout. However, real‐time sensors can record the agonist‐induced fluctuation of signal from the same cells; therefore, even a low percentage of signal change could potentially be detected if the background of the system is low. Integration sensors require higher SNRs than real‐time sensors, because their agonist‐induced signal changes are not compared within the same cell but, instead, between different animals treated under different conditions. Typically, a minimum SNR of 5 or higher is required for integration reporters. Additionally, due to the protein level‐dependence for integration sensors, single‐component integration reporters are less variable and more advantageous than multi‐component integration reporters.

Due to their complementary strengths and limitations, both real‐time and integration sensors are useful for studying endogenous GPCR agonist release and their effects on neuromodulation and other physiological processes. Genetically encoded real‐time sensors of GPCR agonists are useful for interrogating the spatiotemporal dynamics of GPCR signalling. However, real‐time imaging does not allow further interrogation of the cell population of interest. Cellular resolution integrators are useful for mapping GPCR agonist release in a large area, being able to interrogate whole‐brain neuronal circuitry and label neurons exposed to the agonists for further interrogation. Integration sensors can be used first for unbiased search of the neuronal circuits affected by endogenous GPCR agonists across the brain. Real‐time sensors can then be used to perform detailed characterisations of the spatiotemporal dynamics of endogenous GPCR agonist release in those circuits. The combined use of these sensors, therefore, can help expand our overall knowledge of GPCR‐mediated neuronal signalling.

## CONFLICT OF INTEREST

The authors declare they have no conflicts of interest.
